# A Case of Multiple Vein Thrombosis Following Pacemaker Implantation

**DOI:** 10.7759/cureus.47466

**Published:** 2023-10-22

**Authors:** Ashwin Jagadish, Abhijith Paladugula, Aditya Thakkar, Debalina Das

**Affiliations:** 1 Internal Medicine, East Tennessee State University James H. Quillen College of Medicine, Johnson City, USA

**Keywords:** duplex ultrasonography, veins, upper extremity deep venous thrombosis, deep vein thrombosis (dvt), pacemaker complication

## Abstract

Pacemakers are implanted as part of the treatment process of conditions including symptomatic bradycardia and certain types of heart block. One complication associated with pacemaker implantation is upper extremity deep venous thrombosis (UEDVT), which can subsequently lead to pulmonary embolism, limb loss, or death. We present the case of an 88-year-old male who developed UEDVT in his left subclavian, axillary, brachial, and basilic veins shortly after dual chamber pacemaker implantation for treating symptomatic heart block. The patient received anticoagulation with intravenous heparin while inpatient but was switched to oral apixaban prior to discharge. This case highlights the importance of detecting and treating UEDVT in patients who recently underwent pacemaker placement.

## Introduction

Pacemakers are part of the treatment plan for conditions such as symptomatic bradycardia, Mobitz type II heart block, and third-degree heart block [1]. Complications of pacemaker implantation may include infection, pericarditis, pneumothorax, hematoma, skin damage, apparatus malfunction, or upper extremity venous thrombosis (UEDVT) [1,2]. UEDVT is reported to be 5-10% of all cases of deep venous thrombosis [3]. Direct oral anticoagulation agents can be used in the management of UEDVT [4].

## Case presentation

An 88-year-old male with a past medical history of hypertension, benign prostatic hyperplasia, gastroesophageal reflux disease, prediabetes, and vitamin D deficiency presented to his outpatient primary care provider (PCP) with new-onset shortness of breath on exertion. Examination revealed bradycardia and electrocardiogram was notable for complete heart block. His most recent stress test was reviewed, revealing low risk and no signs of ischemia. He was advised to proceed to the emergency department for further management. 

**Figure 1:**
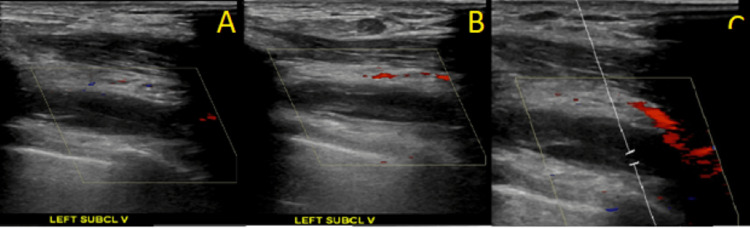
Ultrasonography of the Left Subclavian Vein A: Ultrasonography demonstrating thrombus of the left subclavian vein. B: Ultrasonography demonstrating lack of compression of the left subclavian vein. C. Ultrasonography demonstrating lack of color flow within the left subclavian vein.

**Figure 2:**
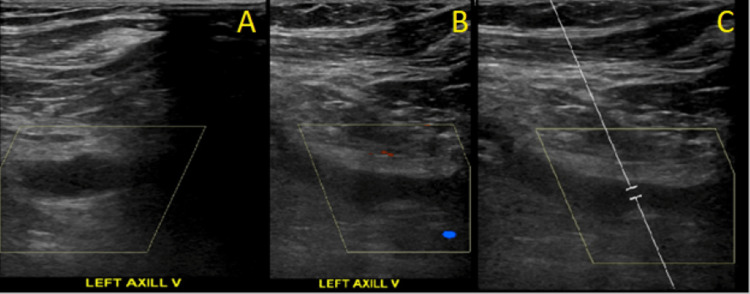
Ultrasonography of the Left Axillary Vein A: Ultrasonography demonstrating thrombus of the left axillary vein. B: Ultrasonography demonstrating lack of compression of the left axillary vein. C. Ultrasonography demonstrating lack of color flow within the left axillary vein.

**Figure 3:**
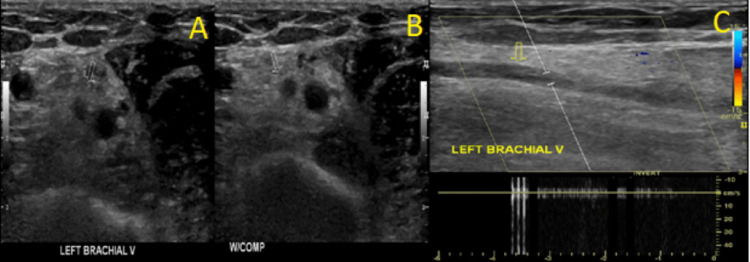
Ultrasonography of the Left Brachial Vein A: Ultrasonography demonstrating thrombus of the left brachial vein. B: Ultrasonography demonstrating lack of compression of the left brachial vein. C. Ultrasonography demonstrating lack of color flow within the left brachial vein.

**Figure 4:**
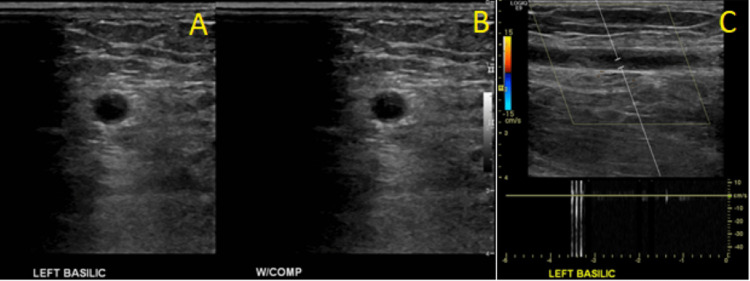
Figure 4: Ultrasonography of the Left Basilic Vein A: Ultrasonography demonstrating thrombus of the left basilic vein. B: Ultrasonography demonstrating lack of compression of the left basilic vein. C. Ultrasonography demonstrating lack of color flow within the left basilic vein.

In the hospital, his echocardiogram revealed a normal left ventricular ejection fraction. He then underwent temporary pacemaker placement, with lead insertion through the right internal jugular vein. The next day, a left-sided dual-chamber pacemaker was successfully implanted without procedural complications, followed by the removal of the temporary pacemaker. The patient was discharged from the hospital the following day. Nine days after discharge, he presented to his PCP; on examination, he was noted to have edema, erythema, and tenderness in his left upper extremity. The patient noted that the swelling began two days after pacemaker placement. Throughout the days following discharge, he did not have fever, chills, erythema around the pacemaker site, or drainage from the pacemaker site. Left venous duplex compression ultrasonography of the extremity was conducted and demonstrated thrombosis of the left subclavian, axillary, brachial, and basilic veins (Figures 1-4). As a result, the patient was advised to go to the emergency department for treatment.

While in the hospital, the patient was started on intravenous heparin. Cardiology and Interventional Radiology evaluated him, and they both recommended pursuing oral anticoagulation rather than thrombectomy. Prior to discharge, he was switched from intravenous heparin to oral apixaban.

## Discussion

Our patient initially presented to his PCP, where he was found to be bradycardic and have complete heart block. He was subsequently referred to the hospital, where he ultimately received implantation of a dual-chamber permanent pacemaker. Nine days after discharge, the patient once again presented to his PCP; an ultrasound was obtained and revealed thrombosis of the left subclavian, axillary, brachial, and basilic veins. He was subsequently admitted to the hospital where he received intravenous anticoagulation, which was replaced by oral apixaban prior to discharge.

Common presenting symptoms of UEDVT include swelling, pain, and erythema of the affected arm [3]. A more serious complication of this condition is phlegmasia cerulea dolens [4]. The level of symptoms may correspond with the degree of venous obstruction [4]. A physical examination focusing on the upper extremities can help narrow the list of differential diagnoses, and guide the provider to the diagnosis of UEDVT [3]. Confirmation of UEDVT is usually conducted using venous duplex compression ultrasonography, a test with 97% sensitivity and 96% specificity [3].

Utilization of transvenous cardiac device leads is an important risk factor for the development of UEDVT, which may occur up to many years after initial device implantation [5]. Within one year of pacemaker placement, UEDVT may be detected by Doppler ultrasound in 23% of patients [6]. Venous duplex ultrasound can show total or partial occlusion of lumen by thrombus, lack of compressibility of the vein, or lack of color flow [4]. Grayscale images may show variable echogenicity of the thrombus in deep veins [7].

Management of UEDVT typically involves anticoagulation [4]. Anticoagulant therapies that may be used include either direct anticoagulant agents, agents that are antagonists of vitamin K, or low molecular weight heparin [4]. Thrombolysis can be considered in severe cases, but anticoagulation alone is typically preferred [4]. 

## Conclusions

Individuals with symptomatic bradycardia and complete heart block may require permanent pacemaker implantation. A known complication of this procedure is the formation of UEDVT. Diagnosis depends on comprehensive history and physical examination and can be confirmed with venous duplex compression ultrasonography. Once diagnosis is made, appropriate anticoagulation therapy can be initiated to treat the condition. 

## References

[1] Puette JA, Malek R, Ellison MB (2022). Pacemaker. StatPearls [Internet].

[2] Basar N, Cagli K, Basar O (2010). Upper-extremity deep vein thrombosis and downhill esophageal varices caused by long-term pacemaker implantation. Tex Heart Inst J.

[3] Grigorian A, Nahmias JT (2023). Upper extremity deep venous thrombosis. StatPearls [Internet].

[4] Mintz A, Levy M (2023). Upper Extremity Deep Vein Thrombosis. American College of Cardiology.

[5] Duijzer D, de Winter MA, Nijkeuter M, Tuinenburg AE, Westerink J (2021). Upper extremity deep vein thrombosis and asymptomatic vein occlusion in patients with transvenous leads: a systematic review and meta-analysis. Front Cardiovasc Med.

[6] van Rooden CJ, Molhoek SG, Rosendaal FR, Schalij MJ, Meinders AE, Huisman MV (2004). Incidence and risk factors of early venous thrombosis associated with permanent pacemaker leads. J Cardiovasc Electrophysiol.

[7] Sosthène TV, Serge KK, Medard KK, Simplice KV, John MK, Albin SS (2020). Duplex ultrasound in upper and lower limb deep venous thrombosis. Ann Circ.

